# Circulating tumour DNA as biomarker for rectal cancer: A systematic review and meta-analyses

**DOI:** 10.3389/fonc.2023.1083285

**Published:** 2023-01-30

**Authors:** Jan M. van Rees, Lissa Wullaert, Alexander A. J. Grüter, Yassmina Derraze, Pieter J. Tanis, Henk M. W. Verheul, John W. M. Martens, Saskia M. Wilting, Geraldine Vink, Jeroen L. A. van Vugt, Nick Beije, Cornelis Verhoef

**Affiliations:** ^1^ Department of Surgical Oncology and Gastrointestinal Surgery, Erasmus MC Cancer Institute, Rotterdam, Netherlands; ^2^ Department of Surgery, Amsterdam University Medical Centres (UMC), Vrije Universiteit Amsterdam, Department of Surgery, Cancer Center Amsterdam, Amsterdam, Netherlands; ^3^ Department of Medical Oncology, Erasmus MC Cancer Institute, Rotterdam, Netherlands; ^4^ Department of Medical Oncology, University Medical Center Utrecht, Utrecht University, Utrecht, Netherlands; ^5^ Department of Research and Development, Netherlands Comprehensive Cancer Organisation, Utrecht, Netherlands

**Keywords:** Ctdna (circulating tumour DNA), cfDNA (circulating free DNA), rectal cancer, minimal residual disease (MRD), liquid biopsy

## Abstract

**Background:**

Circulating tumour DNA (ctDNA) has been established as a promising (prognostic) biomarker with the potential to personalise treatment in cancer patients. The objective of this systematic review is to provide an overview of the current literature and the future perspectives of ctDNA in non-metastatic rectal cancer.

**Methods:**

A comprehensive search for studies published prior to the 4^th^ of October 2022 was conducted in Embase, Medline, Cochrane, Google scholar, and Web of Science. Only peer-reviewed original articles and ongoing clinical trials investigating the association between ctDNA and oncological outcomes in non-metastatic rectal cancer patients were included. Meta-analyses were performed to pool hazard ratios (HR) for recurrence-free survival (RFS).

**Results:**

A total of 291 unique records were screened, of which 261 were original publications and 30 ongoing trials. Nineteen original publications were reviewed and discussed, of which seven provided sufficient data for meta-analyses on the association between the presence of post-treatment ctDNA and RFS. Results of the meta-analyses demonstrated that ctDNA analysis can be used to stratify patients into very high and low risk groups for recurrence, especially when detected after neoadjuvant treatment (HR for RFS: 9.3 [4.6 – 18.8]) and after surgery (HR for RFS: 15.5 [8.2 – 29.3]). Studies investigated different types of assays and used various techniques for the detection and quantification of ctDNA.

**Conclusions:**

This literature overview and meta-analyses provide evidence for the strong association between ctDNA and recurrent disease. Future research should focus on the feasibility of ctDNA-guided treatment and follow-up strategies in rectal cancer. A blueprint for agreed-upon timing, preprocessing, and assay techniques is needed to empower adaptation of ctDNA into daily practice.

## Introduction

Rectal cancer is a worldwide cause of cancer-related mortality, with a global incidence of approximately 732,200 new cases per year (1). The introduction of combined neoadjuvant (chemo)radiotherapy and total mesorectal excision (TME) has significantly reduced the local recurrence rate, though distant recurrence rates remain around 30% (2). Recurrences are likely to derive from residual locoregional disease after surgery or subclinical metastatic disease (minimal residual disease) (3). These micrometastases are undetectable by the currently used imaging techniques. Carcinoembryonic antigen (CEA) is a widely accepted tumour marker in the follow-up of colorectal cancer, but is imperfect due to the limited accuracy of this test to detect recurrence, mostly owing to its high rate of false positive results (4, 5). Consequently, there is an urgent need for novel techniques to detect minimal residual disease after standard treatment, in order to identify those patients who are at high risk for recurrent disease.

Classification of these patients would enable a ‘tailored’ postoperative treatment approach, in which patients could be stratified into groups who may benefit from additional treatment or, otherwise, less intensive surveillance.

Circulating tumour DNA (ctDNA) is a component of the total amount of cell-free DNA (cfDNA), and it presumed that this ctDNA is shed into the bloodstream by necrotising cancer cells. Measurement of ctDNA in peripheral blood samples has been established as a promising biomarker, with the potential to optimise tailored treatment in cancer patients (6–8). In recent years, ctDNA has been investigated in various cancer types and settings, and is considered to be an important diagnostic tool for the detection of minimal residual disease after surgery. The potential clinical utility of ctDNA has already been established in certain fields. In stage II colon cancer, ctDNA-guided treatment resulted in a reduction in the number of patients receiving adjuvant therapy when compared to conventional stratification methods, whilst not altering the risk of recurrence (9). For rectal cancer, research establishing the true clinical value of ctDNA-guided treatment has yet to be conducted. In addition, there is still a lack of consensus whether the use of adjuvant chemotherapy is justified in rectal cancer patients, and postoperative treatment regimens differ per country (10, 11).

During curative treatment of rectal cancer, there are several methods and time points when ctDNA could be measured in peripheral blood samples. At diagnosis and before any treatment, the amount of ctDNA could be associated with the extent of the disease. During or after neoadjuvant treatment, changes in the level ctDNA could be associated with response or progression. Finally, the presence of ctDNA after surgery is an indication of minimal residual disease. The conceivable added value of ctDNA in rectal cancer is its potential application as a guide for therapy selection. Herein, patients who are stratified as high-risk for recurrence could, for example, be treated with adjuvant systemic therapy, while patients without detectable ctDNA after neoadjuvant treatment and surgery might be suitable for less intensive follow-up regimes.

In literature, several methods have been described to analyse the presence of ctDNA in peripheral blood samples, with different recommendations regarding pre-analytical conditions (12–14). In rectal cancer, two main ctDNA detection techniques are measuring the absolute number of cfDNA or identifying tumour-specific somatic mutations (15). These mutations are usually detected using polymerase chain reaction (PCR) or next-generation sequencing (NGS). Although PCR is a viable option to detect a small number of already known somatic mutations, the main advantage of NGS is the possibility to interrogate multiple genes at once, and it does not necessarily require prior knowledge of a specific mutation profile. Both techniques could either be applied to the unique mutations of the patient’s tumour (i.e., *tumour-informed with specific panel*) or to a universal panel of genes commonly mutated in (colorectal) cancer patients (i.e., *tumour-agnostic*). Finally, a universal panel could be used that is evaluated by the patients’ tumour tissue (i.e., *tumour-informed with predefined panel*). Given the heterogeneity in measurement techniques of ctDNA, a summary of the applied techniques in previous studies may provide insight in suitable approaches for specific purposes.

The aim of this literature review is to provide an overview of the current evidence and ongoing trials in the field of ctDNA in non-metastatic rectal cancer.

## Methods

This systematic review and meta-analyses were conducted according to the PRISMA guidelines (Preferred Reporting Items for Systematic Reviews and Meta-analysis). A comprehensive search was performed in five databases (Embase, Medline, Cochrane, Web of Science and Google Scholar), including potential studies published prior to the 4^th^ of October 2022. Only English-written, peer-reviewed clinical studies that investigated the association between ctDNA and oncologic outcomes in non-metastatic rectal cancer patients were included. Non-original articles (i.e. review articles and meta-analyses) and case reports were excluded. The complete search term performed on the 4^th^ of October 2022 is shown in **Supplementary 1**.

### Study selection and quality assessment

Screening of the articles was performed by two independent authors (JR, LW) and disagreement was resolved through joint assessment and in collaboration with a third reviewer (NB). Quality assurance was performed by two individual reviewers (JR, LW) according to the Quality In Prognosis Studies tool (QUIPS) (16). Three categories of risk of bias were considered as the outcome of the QUIPS tool, being low, moderate and high risk of bias. The outcomes of the quality assessment using the QUIPS tool were visualised using the Risk-of-bias VISualization (robvis) tool (17). In case of disagreement, joint evaluation was performed, and a third reviewer (SW) was approached when deemed necessary. Study characteristics like study design, sample size and specifications about the ctDNA assessment (collection time points, target, assay type, quantification method, whether the technique was NSG or PCR based and whether it was tumour informed) were collected.

### Meta-analyses

Meta-analyses were performed using the generic inverse-variance method using a random-effects model. Herein, only studies that reported hazard ratios with either confidence intervals or p-values, for recurrence-free survival (RFS) or disease-free survival (DFS) were included. Studies that did not report appropriate or sufficient data for the pooled analysis were separately discussed. Outcomes of interest included: hazard ratios (HR), 95% confidence intervals (CI), I^2^ values for heterogeneity, and p-values, in which a value <0.05 was considered statistically significant. Meta-analyses and figures were established from Review Manager (RevMan) version 5.4.1, The Cochrane Collaboration, 2020.

## Results

**Figure 1:**
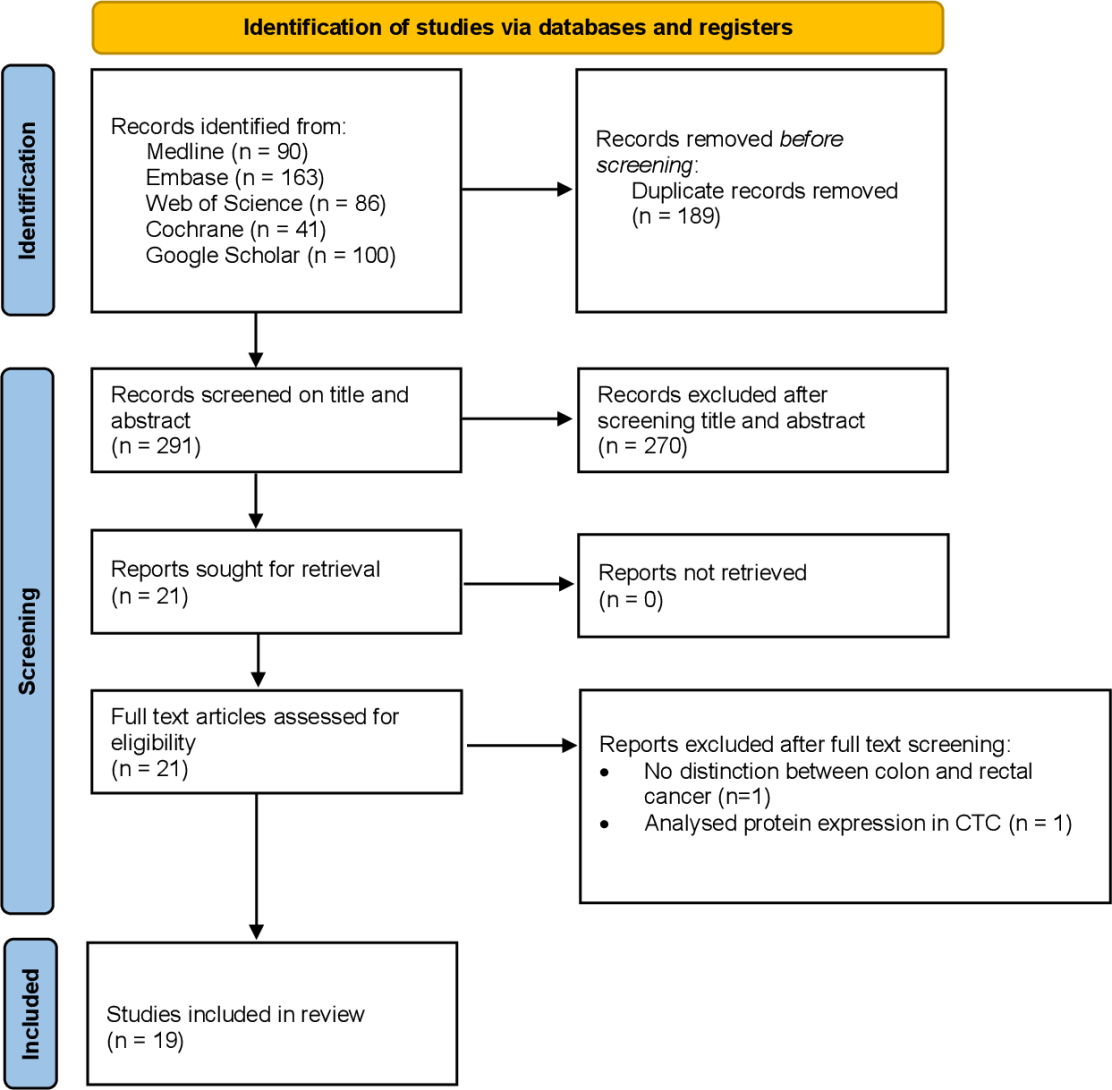
PRISMA flowchart.

A total of 480 records were retrieved by the systematic search, of which 189 were duplicates, 261 were original publications and 30 were ongoing trials (**Figure 1**). All 291 unique studies and trials were screened for eligibility, after which 270 publications were excluded by reading title and abstract. Reasons for exclusion were reports of conference abstracts, case reports, (systematic) reviews, studies that did not include patients with rectal cancer, and studies that had not investigated clinical outcomes. The full text of twenty-one studies was assessed, of which two additional studies were excluded due to a lack of distinction between colon and rectal cancer, and due to an analysis of circulating tumour cells, which was ineligible for the current meta-analysis. A total of nineteen studies is discussed in this literature review, of which seven were included in the meta-analysis. For each included study a quality assurance was performed according to the QUIPS tool, as shown in **Supplementary 2**. Study characteristics, including outcome measures and the number of patients, are reported in **Table 1**.

**Table 1:** Study characteristics.

Author, year	Study design	Patients	Assay type	NSG / PCR	Tumour informed	Time points (s)	Outcome (binary)	Risk of Bias
Zitt et al. (18)	Prospective cohort, single centre	LARC, 26	cfDNA concentration	PCR	Agnostic	BL, post-CRT, end treatment	Treatment response	High
Agostini et al. (19)	Prospective pilot study	LARC, 67	cfDNA concentration	PCR	Agnostic	BL, post-CRT	Treatment response	High
Sun et al. (20)	Prospective cohort, single centre	LARC, 34	Multiple	PCR	Agnostic	BL, post-CRT	Treatment response	High
Boysen et al. (21)	Retrospective cohort	LARC, 75	cfDNA concentration	PCR	Agnostic	Post-CRT	Both	High
Liu et al. (22)	Prospective cohort, multicentre	LARC, 82	Mutation-specific panel	NGS	Both	During and post-NAT	Long-term (oncologic) survival	Low
Sclafani et al. (23)	Prospective cohort, multicentre	LARC, 97	Mutation-specific panel	PCR	Tumour informed (predefined panel)	BL	Both	High
Schou et al. (24)	Prospective cohort, single centre	LARC, 123	cfDNA concentration	dFA	Agnostic	BL, after induction chemotherapy, after CRT, serial samples 5 years after surgery	Long-term (oncologic) survival	High
Tie et al. (25)	Prospective cohort, multicentre	LARC, 159	Mutation-specific panel	NGS	Tumour informed (tumour specific)	BL, post-CRT, post-surgery	Long-term (oncologic) survival	Low
Appelt et al. (26)	Prospective cohort, multicentre	LARC, 146	cfDNA concentration	PCR	Agnostic	BL	Long-term (oncologic) survival	High
Guo et al. (27)	Unknown	LARC, 194	Promoter genes	NGS	Agnostic	BL	Treatment response	High
Khakoo et al. (28)	Prospective cohort, single centre	LARC, 47	Mutation-specific panel	PCR	Tumour informed (tumour specific)	BL, mid CRT, post-CRT, after surgery	Both	Low
Murahashi et al. (29)	Prospective cohort, single centre	LARC, 85	Mutation-specific panel	NGS	Agnostic	BL, post-NAT, post-surgery	Both	Moderate
Pazdirek et al. (30)	Prospective cohort, single centre	LARC, 36	Mutation-specific panel	PCR	Tumour informed (predefined panel)	BL, during CRTx	Long-term (oncologic) survival	Moderate
Zhou et al. (31)	Prospective cohort, multicentre	LARC, 106	Mutation-specific panel	NGS	Tumour informed (tumour specific)	BL, during CRT, presurgery, and postsurgery	Long-term (oncologic) survival	Low
McDuff et al. (32)	Retrospective cohort	LARC, 29	Mutation-specific panel	PCR	Tumour informed (tumour specific)	BL, preoperatively, and postoperatively	Both	Moderate
Vidal et al. (33)	Prospective cohort, multicentre	LARC, 119	Mutation-specific panel	NGS	Agnostic	BL, during nCRT, and after surgery	Both	Moderate
Wang et al. (34)	Prospective cohort, single centre	LARC, 72	Mutation-specific panel	NGS	Tumour informed (predefined panel)	BL, post-NAT	Both	Low
Roesel et al. (35)	Prospective cohort, multicentre	LARC, 25	Mutation-specific panel	NGS	Tumour informed (predefined panel)	T0: first day of radiotherapy Tend: last day of radiotherapy T4: 4 weeks after radiotherapy T7: 7 weeks after radiotherapy Top: day of surgery Tpost-op: 3-7 days after surgery TIMV: mesenteric vein sample during surgery	Treatment response	Low
Truelsen et al. (36)	Prospective cohort, single centre	LARC, 76	cfDNA concentration	dFA	Agnostic	BL, mid therapy and at end of therapy	Treatment response	High

BL, baseline; cfDNA, cell-free DNA; CRT, chemoradiotherapy; dFA, direct fluorescence assay; LARC, locally advanced rectal cancer; NAT, neoadjuvant treatment; NSG, next generation sequencing; PCR, polymerase chain reaction.

Nine out of nineteen (47%) included studies were considered high risk of bias, six (32%) received a low risk of bias score, and four studies (22%) a moderate risk of bias. High risk of bias was mostly due to bias in prognostic factor measurement and attrition, as depicted in the graph in **Supplementary 3**. ctDNA measurement techniques varied greatly among included studies. Most frequently used quantification methods were digital droplet PCR (ddPCR), real time PCR (qRT-PCR) and next generation sequencing (NGS). Five studies designed their panel based on the unique tumour and patient (tumour informed – tumour specific). Four studies applied a tumour informed predefined panel, and ten adopted a tumour agnostic approach. Liu et al. investigated multiple ctDNA techniques (22). All studies in this review only included patients with locally advanced rectal cancer (LARC). No eligible studies were found that included non-LARC patients.

### Original articles

All included studies were either prospective or retrospective cohort studies. A total of 1598 patients undergoing treatment for LARC were included, with sample sizes ranging from 25 to 159 patients. The methods for ctDNA analyses (assay type, quantification method, tumour-informed or -agnostic) are described in **Table 1**. Twelve studies (63%) used a mutation-specific panel, of which nine were tumour-informed. Seven other studies measured total cfDNA concentration. Nine studies quantified ctDNA with a PCR-based technique. NGS was the chosen technique in eight studies, and another two studies used the direct fluorescent assay (dFA). Time points at which ctDNA was measured varied, and are reported from baseline (defined as before the start of any treatment) up until last follow-up after definite treatment. Additional details regarding plasma isolation, cfDNA isolation, and pre-processing conditions can be found in **Supplementary 4**.

### ctDNA and treatment outcomes in rectal cancer (cfDNA concentration studies)

The earliest study in the systematic search reporting clinical outcomes, published in 2008, investigated changes in cfDNA levels before and after neoadjuvant chemoradiation in patients with LARC using quantitative real-time polymerase chain reaction (qRT-PCR) (18). No association was found between baseline cfDNA levels and tumour response, but the study showed that patients who responded to chemoradiation had a decrease in cfDNA levels (median 2.2 ng/mL), whereas in patients without response, cfDNA levels significantly increased (median 5.1 ng/mL) (P = 0.006). The authors concluded that cfDNA concentration could be used for therapy monitoring in patients with rectal cancer undergoing preoperative chemoradiation, and these findings were repeatedly confirmed in several other exploratory studies (19–21, 36).

### ctDNA and long-term oncologic survival outcomes in rectal cancer (cfDNA concentration studies)

Besides the use of cfDNA for response outcomes, cfDNA was investigated as predictor for long-term (oncological) outcomes as well. In 2017, Boysen et al. were the first to find an association between the level of pre-surgery cfDNA and the risk of recurrence after surgery (21). In this study including 75 patients with LARC, the level of cfDNA was quantified by ddPCR and expressed as copy number of beta 2 microglobulin. The median levels of cfDNA for patients with recurrent disease were 13,000 copies/mL compared to 5200 copies/mL for non-recurrent patients (p = 0.08).

In line with this, Schou et al. demonstrated, in a study with 123 participants, that patients with baseline cfDNA levels above the 75th quartile measured by a direct fluorescent assay, had a higher risk of local or distant recurrence and shorter time to recurrence compared with patients with plasma cfDNA below the 75th percentile (HR = 2.48, 95% CI: 1.3–4.8, P = 0.007) (24). The same applied to DFS (HR = 2.43, 95% CI: 1.27–4.7, P = 0.015). In a subgroup analysis with 71 patients who received induction chemotherapy (capecitabine and oxaliplatin (CAPOX)) before chemoradiation, the prognostic impact of plasma levels of cfDNA remained significant for time to recurrence and DFS. In multivariate analysis, a high cfDNA level was significantly associated with time to progression and DFS. During follow-up, the association remained significant regardless of time point for sample analysis.

Finally, Appelt et al. found that fractional abundance of hypermethylation of the neuropeptide Y gene in cfDNA (meth-cfDNA), could be used as baseline prognostic marker as well (26). They showed in 146 LARC patients that meth-cfDNA, determined by quantitative PCR on baseline, was associated with a significantly worse overall survival (adjusted HR: 2.08, 95% CI: 1.23-1.51) and distant metastases rate (55% *vs*. 72% at 5 y, p=0.01).

### ctDNA and long-term oncologic survival outcomes in rectal cancer (mutation-specific assay studies)

While multiple studies described the prognostic value of cfDNA concentrations, an important downside is that these assays lack the ability to discriminate between cfDNA from healthy cells and cfDNA directly derived from the tumour (ctDNA). Especially in the context of MRD detection, there is a need for tests with high specificity.

Therefore, in recent years, more and more studies utilising techniques that can specifically detect ctDNA have increasingly been described (22, 25, 28–35). The largest study conducted so far by Tie et al., including 159 patients with LARC, has demonstrated that ctDNA status could be used to classify groups as very high and low risk for recurrence (25). Somatic mutations in individual patient’s tumours were identified *via* massively parallel sequencing of 15 genes commonly mutated in colorectal cancer, after which personalised assays were designed to quantify ctDNA in plasma samples. Prior to neoadjuvant (chemo)radiotherapy 122 (77%) patients had detectable ctDNA. After surgery, 19 patients (12%) had detectable ctDNA of which 58% recurred during follow-up (median 24 months). In contrast, recurrence occurred in only 8.6% of the patients without detectable ctDNA (HR 13, 95% CI 5.5-31, p<0.001). The prognostic value of detectable ctDNA for recurrence was even stronger in patients with a high pathological stage (ypT3-4 and ypN1-2), demonstrated by recurrence rates up to 89% after 2 years in patients with detectable ctDNA after surgery combined with pathologically staged lymph node metastases. This study also showed that the predictive value of ctDNA was strong when measured after treatment. No difference in RFS was observed between patients with detectable ctDNA and those without detectable ctDNA before treatment (HR 1.1; 95% CI: 0.42 - 3.0). However, for the post-treatment measurements, the Kaplan-Meier estimates of RFS at 3 years were 50% (95% CI: 28% - 88%) and 85% (95% CI: 79% - 93%) for the postchemoradiation ctDNA-positive and ctDNA-negative groups respectively, and 33% (95% CI: 16% - 72%) and 87% (95% CI: 79% - 95%) for the postoperative ctDNA-positive and ctDNA-negative groups. This study also demonstrated that postoperative CEA (≥5.0 ng/ml) was also a predictor for recurrence (adjusted HR 5.1, 95% CI: 1.3 - 18), but that in patients with normal CEA, postoperative detectable ctDNA remained associated with a high risk of recurrence (HR 8.8, 95% CI 3.2 – 24; P<0.001).

Another prospective multicentre study also investigated the predictive value of ctDNA analysed by targeted NGS at different time points before and during treatment in 106 LARC patients undergoing chemoradiation (31). Mutations in cfDNA were only called as somatic mutations if these mutations were also present in the primary tumour, which was also subjected to targeted NGS. ctDNA was detected in 75% of patients at baseline, 16% during chemoradiation, 11% before surgery, and 7% after surgery. Again, detectable ctDNA after surgery was the strongest predictive factor for distant metastasis (HR 25.30, 95% CI 1.475-434.0), compared to one cycle after the initiation of chemoradiation (HR 6.635, 95% CI: 1.240-35.50), and 7 weeks after chemoradiation (before surgery) (HR 19.82, 95% CI: 2.029-193.7). However, these subgroup analyses were underpowered (only 6 patients had detectable ctDNA in the postoperative ctDNA group).

Khakoo et al. investigated the role of ctDNA by tracking up to three somatic variants that were found in tumour tissue in plasma using ddPCR in patients with LARC (28). They showed that all three patients with detectable ctDNA after surgery had recurrent disease compared with none of the 20 patients with undetectable ctDNA (P = 0.001). Similar results were found in a study conducted by McDuff et al. (32) In this study, NGS was used to identify mutations in the primary tumour, and mutation-specific ddPCR were used to assess mutation fraction in ctDNA. The study found that all four LARC patients with detectable postoperative ctDNA recurred (positive predictive value = 100%), whereas only two of 15 patients with undetectable ctDNA recurred (negative predictive value: 87%). The hazard ratio for RFS at a median follow-up of 20 month was 12 in patients with detectable postoperative ctDNA (P = 0.007). Another study of 119 LARC patients demonstrated that post-operative ctDNA testing with a tumour-agnostic customised NGS panel targeting 422 cancer-related genes, in combination with a high-risk pathological feature (perineural invasion, tumour deposits, vascular invasion, and lymph node metastasis), was able to predict the recurrence of all six patients that were analysed in this risk group (HR 90, 95% CI: 17 – 479 compared to undetectable ctDNA and no high risk features) (34).

Another prospective cohort study conducted by Murahashi et al. used NGS on a cfDNA panel with 14 target genes to investigate the association of ctDNA on preoperative treatment response and postoperative recurrence in 85 LARC patients (29). A significant association was found between changes in ctDNA before and after neoadjuvant treatment (≥80% change in cfDNA versus < 80% change in cfDNA) and pathological complete response (OR 8.5; 95% CI: 1.4–163). In addition, the rate of recurrent disease was significantly higher in patients with high levels of postoperative ctDNA (≥0.5%) than in those with low levels of ctDNA (<0.5%) (HR 17.1, 95% CI: 1.0-282). In this study, postoperative CEA (≥5.0 ng/ml) was also independently associated with recurrence (adjusted HR: 6.9, 95% CI 1.6–29), and all four patients that had a combination of detectable ctDNA and CEA had disease relapse (HR: 34, 95% CI: 0.4 - 2631).

The phase II GEMCAD 1402 study, including 72 patients with LARC undergoing total neoadjuvant treatment (fluorouracil, leucovorin, and oxaliplatin with or without aflibercept, followed by chemoradiation and surgery), also evaluated ctDNA as biomarker to predict tumour response and survival outcome (33). ctDNA was detectable using a tumour-agnostic CRC-specific NGS assay (Guardant reveal) integrating somatic mutations and epigenomic signatures in 83% of patients at baseline and in 15% following total neoadjuvant treatment (pre-surgery). Baseline ctDNA detection was not associated with poor survival outcomes, but detectable ctDNA just before surgery (after total neoadjuvant treatment) was significantly associated with systemic recurrence, shorter DFS (HR, 4; P = 0.033), and shorter overall survival (HR, 23; P < 0.0001). The predictive value of detectable ctDNA after surgery was not investigated in this study.

Finally, an exploratory study by Liu et al. analysed three different ctDNA techniques in LARC patients in samples taken after neoadjuvant treatment (22). The three ctDNA assays were: 1. a tumour-informed personalized assay, 2. a tumour-agnostic targeted assay of genes frequently mutated in CRC, and 3. a copy number alteration-based approach. All three investigated techniques were associated with a poor RFS. The personalised assay targeting tumour-informed mutations was significantly associated with an increased risk of recurrence (HR = 27.38; log-rank P < 0.0001), the universal panel of genes frequently mutated in colorectal cancer (HR = 5.18; log-rank P = 0.00086), and the low depth sequencing for copy number alterations (CNAs) analysis showed a compromised performance in predicting recurrence (HR = 9.24; log-rank P = 0.00017). Of note, this study was not powered to detect differences between the three assays.

### Alternative cfDNA and ctDNA techniques

Alternative methods to enable the use of cfDNA in clinical practice have been described as well. Guo et al. analysed gene promoter coverage in cfDNA of 20 patients with LARC (both 10 patients with- and without pathological complete response), in order to predict tumour expression status and subsequently patients’ response to chemoradiation (27). Thus, this study did not investigate mutations (ctDNA), but determined the relative coverage of gene promoter regions in the cfDNA. In a letter to the editor, they propose a classifier of promoters with differential coverage between cfDNA of patients with and without pathological complete response, and validated the use of this prediction technique in 194 LARC patients. The classifier resulted in an AUC of 0.89 (0.83‐0.94) to discriminate patients with and without pathological response, but no external validation of this classifier was performed.

Sclafani et al. used ctDNA to assess KRAS/BRAF mutations in baseline blood samples from 114 patients with LARC, and compared these to mutations in tumour tissue (23). Notably, in 26 patients the ctDNA analysis revealed a KRAS mutation that was not previously found in tumour tissue using standard PCR-based techniques. However, a more sensitive technique (ddPCR) and additional analysis of a different tissue section revealed that 22 of these 26 “newly” detected plasma mutations were already detectable in the tumour in hindsight. In this study, no association between the presence of KRAS/BRAF in ctDNA and clinical outcomes was found.

### Meta-analyses

**Figure 2:**
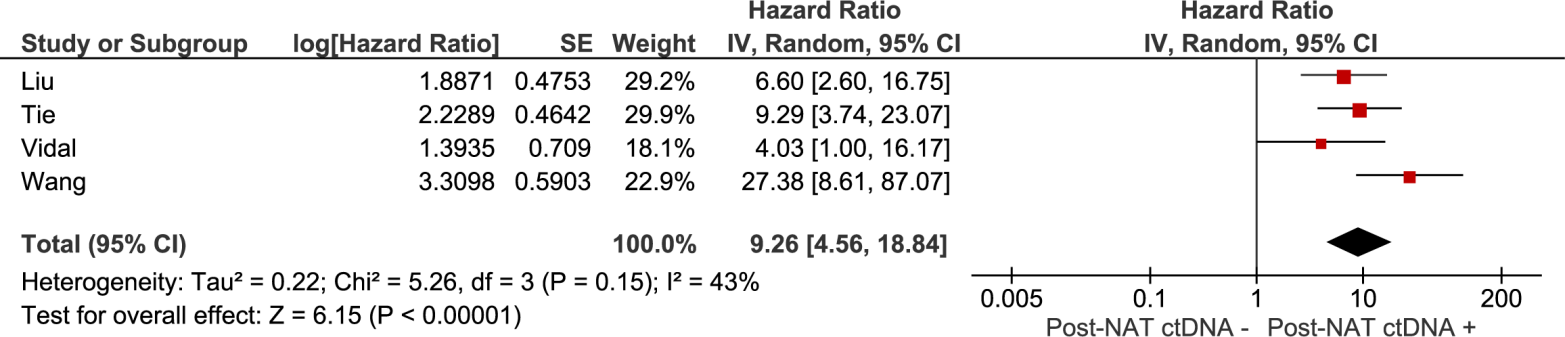
Meta-analysis of the association between recurrence-free survival and the presence of ctDNA after neoadjuvant treatment (chemoradiation with or without systemic treatment).

**Figure 3:**
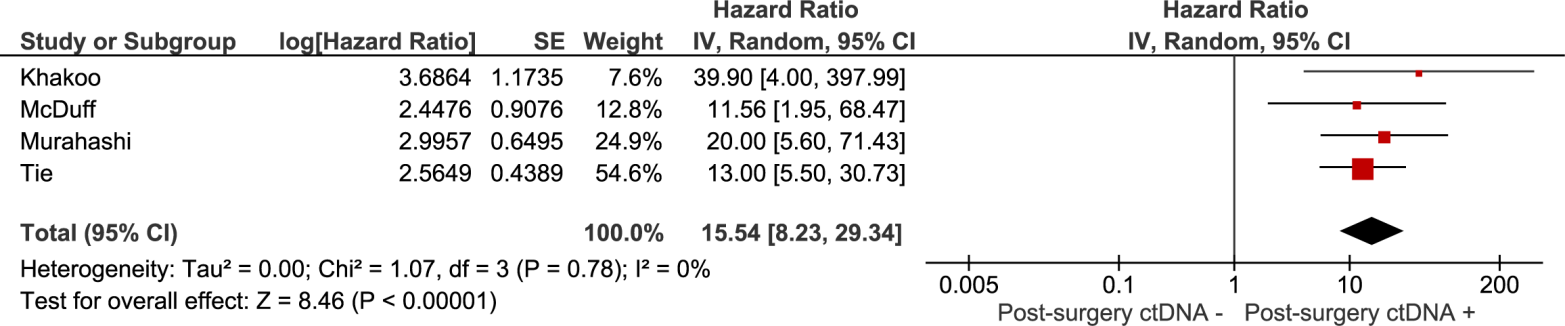
Meta-analysis of the association between recurrence-free survival and the presence of ctDNA after curative intent surgery.

The association between recurrence-free survival and: 1) the presence of ctDNA after neoadjuvant treatment (chemoradiation with or without systemic treatment), 2) the presence of ctDNA after curative intent surgery were investigated in meta-analyses. Results are summarised in **Figures 2**, **3**. The pooled hazard ratio for ctDNA presence after neoadjuvant treatment was 9.26 (95% CI: 4.56 – 18.84) compared to those patients who were without detectable ctDNA after neoadjuvant treatment. After surgery, patients with detectable ctDNA had increased risk for recurrence, compared to patients without detectable ctDNA (HR 15.54, 95% CI: 8.23 – 29.34).

### Ongoing ctDNA trials in rectal cancer

Two interventional trials were found in the systematic search investigating the use of ctDNA in patients with rectal cancer, being the DYNAMIC-RECTAL trial (ACTRN12617001560381) and the SYNCOPE study (NCT04842006). The aim of the DYNAMIC-RECTAL trial was to randomise 408 patients to either a ctDNA-informed arm and a standard of care arm (37). In the ctDNA-informed arm, patients would receive adjuvant chemotherapy if ctDNA was detected, or a not detected in the presence of a high-risk tumour (based on the standard pathology risk assessment of the tumour). In the standard of care arm, the decision regarding adjuvant chemotherapy was based on the standard pathology risk assessment of the tumour. Recruitment of this study terminated early, as accrual slowed down due to the COVID-19 pandemic and the total neoadjuvant treatment approach in this population was adopted. Therefore, the target number could not be reached within the planned recruitment period.

The SYNCOPE study randomises 93 rectal cancer patients into a group of patients that will be treated with novel precision methods, being ctDNA and organoid-guided adjuvant therapy, and a group of patients that will undergo conventional treatment strategy. Primary outcomes are RFS and the number of patients with detectable ctDNA in the postoperative sample of patients in the conventional treatment arm who are not assigned to chemotherapy.

## Discussion

The aim of this literature review was to provide an overview of the current evidence and ongoing trials in the field of ctDNA in non-metastatic rectal cancer. Studies have consistently shown the strong association between detectable ctDNA after treatment and unfavourable prognosis. It can be concluded from these results that ctDNA analysis from peripheral blood samples, especially detected after surgery with curative intent, stratifies patients into two groups: one with a very high risk for recurrence, another with a low risk for recurrence. Thus far, there are no rectal cancer trials published, that have investigated ctDNA-guided adjuvant treatment in a randomised setting.

Based on our systematic search, this systematic review is the first to pool long-term oncological survival outcomes in a meta-analysis. A systematic review by Boyson et al. included nine single arm studies with a total of 615 patients undergoing chemoradiation for rectal cancer and investigated the relation between ctDNA and clinical outcomes (15). Eight of the nine studies showed some degree of correlation between ctDNA and either response to chemoradiation, risk of recurrence or disease-free survival. A second systematic review also included nine studies and investigated the association between clinical outcomes and ctDNA at different time points (at diagnosis, after chemoradiation, and after surgery) (38). No association was found between treatment response and ctDNA status at baseline. Studies reporting the prognostic impact of ctDNA after chemoradiation and before surgery showed varying results. All five studies reporting outcomes of detectable ctDNA postoperative and clinical outcomes, found an association between ctDNA positivity after surgery and worse survival. This review demonstrated that post-operative ctDNA is the most predictive prognostic factor of all investigated time points. A third systematic review investigating different ctDNA measurement techniques on predictive and prognostic outcomes in LARC patients, concluded that detection of ctDNA at different time points of treatment was consistently associated with worse prognosis, but that the ideal method and timing for the liquid biopsy still needed to be defined (39).

Although all studies found a positive correlation between ctDNA and treatment and oncological outcomes, various methods to analyse ctDNA were used, including those with quantitative (e.g. absolute cfDNA concentration) and qualitative (tumour-specific somatic mutations) measurements. Articles that utilized quantitative analyses were generally published between 2008-2018, and were considered relatively inferior because quantitative tests do not have the ability to discriminate tumour DNA from physiological circulating DNA from non-cancerous cells. More recent studies often used qualitative techniques that are able to specifically detect tumour-specific cfDNA. These mutation-specific analyses are nowadays considered as technique of choice, and are acceptable in terms of costs (40). Differences in qualitative analyses exist as well, as was shown as shown by Liu et al. (22) This study revealed that minor differences in the sensitivity of ctDNA are observed when different gene panels and techniques for ctDNA quantification are used, in which a personalised assay targeting tumour-informed mutations was suggested to yield the best performance. However, tumour-informed assays are more expensive and labour-intensive as they require sequencing of the tumour and subsequent design of tumour-specific assays. This can be challenging, especially in a setting where the turnaround time for clinical decision-making needs to be short and will be accompanied by higher costs. A tumour-agnostic method is likely to have a faster turnaround time, as it is easier to conduct, and is accompanied by lower costs. Currently, well-powered studies in a real-world setting comparing all assays with regard to its sensitivity, specificity and turnaround time are lacking.

Another controversy in ctDNA analysis is the optimal timing of measurement to detect MRD after surgery, as it has been suggested that an abundance of surgery‐induced cfDNA fragments could hamper the detection of ctDNA from the tumour (41). In a study by Hendriksen et al., it was shown that cfDNA levels in patients with colorectal cancer were increased by threefold during the first week after surgery (median 3.6‐fold increase, mean: 4.0, 95% CI 2.90–5.37, P = 0.0005), and slowly decreased over the next 3 weeks. Notably, it was assumed that in five of the eight patients, ctDNA was falsely measured as being negative, as these patients were ctDNA positive in all other measurements in which ctDNA surgery‐induced cfDNA fragments were not increased. Therefore, to maximize sensitivity of the measurement, one could argue to only measure ctDNA at least four weeks after surgery. On the other hand, when the results of the ctDNA analyses have clinical consequences, e.g. ctDNA-based adjuvant therapy, results ought to be known within the timeframe that consolidation treatment will still be sufficient. Typically, most ctDNA assays are accompanied by an additional four weeks turnover time from blood withdrawal to definite results (42), so the typical timeframe of a maximum of 8 or 12 weeks from surgery to start with adjuvant treatment could be endangered when delaying the ctDNA result too long (43–45). A balance between test sensitivity, and considerations regarding turnaround times inherent to different methods, should be considered for each clinical implication and setting.

Precision biomarkers to predict postoperative outcomes, such as ctDNA, could contribute to the ongoing debate whether additional treatment should be considered after rectal cancer surgery. The role of adjuvant systemic treatment in rectal cancer has not been established globally; practice differs between Europe and the USA, and between European countries as well. In the Netherlands, adjuvant chemotherapy is not recommended for any stage (46). There are only a few randomised controlled trials on adjuvant chemotherapy for rectal cancer available, which yielded conflicting results (47). The fact that the benefit of adjuvant chemotherapy has not yet been demonstrated, is likely related to a dilution effect, and it might very well be true that a subgroup of patients will benefit from additional treatment. Therefore, it would certainly be of interest to explore whether high-risk patients based on ctDNA detected in postoperative peripheral blood samples might benefit from adjuvant treatment. A trial randomising patients with detectable ctDNA into an adjuvant treatment group and a follow-up group is warranted. Such a trial should be able to answer the important question whether ctDNA-guided adjuvant treatment is beneficial in rectal cancer.

Another potential opportunity of ctDNA-guided treatment is the ability to tailor follow-up strategies based on patients’ individual risk of recurrence. As intensive follow-up does not appear to improve overall and cancer-specific survival and quality of life in colorectal cancer, there seems to be an incentive to reduce surveillance after curative surgery (46, 48, 49). Studies have demonstrated that ctDNA outperforms CEA in (colo)rectal cancer patients to detect relapsing disease (5, 25, 31, 50). Therefore, ctDNA-based risk prediction for recurrence may very well be an excellent biomarker to stratify patients without detectable DNA into a less intensive and decentralised surveillance programme in the home environment or even earlier discharge of standard follow-up. This could eventually improve health-related quality of life, cause a reduction in health-related and societal costs as well as anxiety in cancer patients, without compromising oncological outcomes. Further research would be needed to investigate whether this ctDNA-guided follow-up approach is feasible in rectal cancer.

Finally, novel technical advances highlight the promise of several tumour-agnostic ways to detect ctDNA (i.e. without prior tissue-based information) in the future. For example, recent results highlight the merit of circulating cell free (cf)DNA methylation analyses for both detection and classification of many cancer types, including colorectal cancer (51–54). Next to methylation profiling, recently discovered “fragmentomics” also shows great promise for the sensitive detection of cancer using cfDNA (55–57). Both cfDNA methylation profiling and fragmentomics capture information from a much broader spectrum of the circulating tumour genome, theoretically enabling a higher analytical sensitivity for the detection of minute traces of ctDNA in case of MRD. Supporting this notion, combining features from different molecular levels was shown to have complementary value for MRD detection in colorectal cancer (58).

In conclusion, in rectal cancer patients treated with neoadjuvant treatment and surgery, a very strong association was found between post-treatment detectable ctDNA and recurrent disease as well as overall survival. Randomised controlled trials are needed to investigate whether this ctDNA-informed risk classification could be used during clinical decision making for the purpose of patient-tailored treatment.

## Data availability statement

The original contributions presented in the study are included in the article/**Supplementary Material**. Further inquiries can be directed to the corresponding author.

## Author contributions

All authors have significantly contributed to this work. JR, LW, AG, YD, CV, NB were involved in writing the introduction, the systematic search, and the article selection process. JR, LW, NB, JV, SW conducted the quality assessment of the included articles and methodology. GV, PT, HV, JM, CV were part of the writing committee. The manuscript was drafted by JM, LW, AG, YD, and corrected by NB, JV, SW, GV, PT, HV, JM, CV. Supervision was provided by SW, NB and CV. All authors contributed to the article and approved the submitted version.
