# Supplementary material for: Circulating tumour DNA as biomarker for rectal cancer: A systematic review and meta-analyses
Source: Front Oncol. 2023 Jan 30;13:1083285. doi: 10.3389/fonc.2023.1083285 (PMC9922989; doi:10.3389/fonc.2023.1083285)
Supplement: Supplementary file 2 [file DataSheet_2.docx]

*Supplementary 1 – Search terms*

The following search was performed on the 4^th^ of October 2022:

**Embase**

*('rectum cancer'/exp OR 'rectum carcinoma'/de OR 'rectum resection'/exp OR 'rectum tumor'/exp OR (((rectum OR rectal) NEAR/3 (cancer* OR carcinom* OR tumor* OR tumour* OR neoplas* OR adenocarcinom* OR resect* OR unresect* OR excision*)) OR proctectom* OR LARC):ab,ti,kw) AND ('circulating tumor DNA'/de OR 'DNA determination'/mj/de OR 'circulating free DNA'/de OR (((free* OR circulat*) NEAR/3 (DNA*)) OR ctDNA* OR ct-DNA* OR cfDNA OR cf-DNA*):ab,ti,kw) NOT ([Conference Abstract]/lim OR [Conference Review]/lim)*

**Medline**

*(exp Rectal Neoplasms/ OR exp Proctectomy/ OR (((rectum OR rectal) ADJ3 (cancer* OR carcinom* OR tumor* OR tumour* OR neoplas* OR adenocarcinom* OR resect* OR excision*)) OR proctectom* OR LARC).ab,ti,kf.) AND (Circulating Tumor DNA/ OR *Sequence Analysis, DNA/ OR Cell-Free Nucleic Acids/ OR (((circulat* OR free*) ADJ3 (DNA*)) OR ctDNA* OR ct-DNA* OR cfDNA OR cf-DNA*).ab,ti,kf.) NOT (news OR congres* OR abstract* OR book* OR chapter* OR dissertation abstract*).pt.*

**Cochrane**

*((((rectum OR rectal) NEAR/3 (cancer* OR carcinom* OR tumor* OR tumour* OR neoplas* OR adenocarcinom* OR resect* OR excision*)) OR proctectom*):ab,ti,kw) AND ((((circulat* OR free*) NEAR/3 (DNA*)) OR ctDNA* OR ct-DNA* OR cfDNA OR cf-DNA*):ab,ti,kw) NOT "conference abstract":pt*

**Web of Science**

*TS=(((((rectum OR rectal) NEAR/2 (cancer* OR carcinom* OR tumor* OR tumour* OR neoplas* OR adenocarcinom* OR resect* OR excision*)) OR proctectom*)) AND ((((circulat* OR free*) NEAR/2 (DNA*)) OR ctDNA* OR ct-DNA* OR cfDNA OR cf-DNA*))) NOT DT=(Meeting Abstract OR Meeting Summary)*

**Google Scholar**

*"rectum|rectal cancer|carcinoma|tumor|tumour|neoplasm|adenocarcinoma|resection|excision"|proctectomy "circulating|ct|cf DNA"|"cell free DNA"|ctDNA|cfDNA|"circulating tumor|tumour DNA"|"cell free tumor|tumour DNA"*

*'rectum|rectal cancer|carcinoma|tumor|tumour|neoplasm|adenocarcinoma|resection|excision'|proctectomy 'circulating|ct|cf DNA'|'cell free DNA'|ctDNA|cfDNA|'circulating tumor|tumour DNA'|'cell free tumor|tumour DNA'*

*Supplementary 2 – Quality Assessment QUIPS tool*


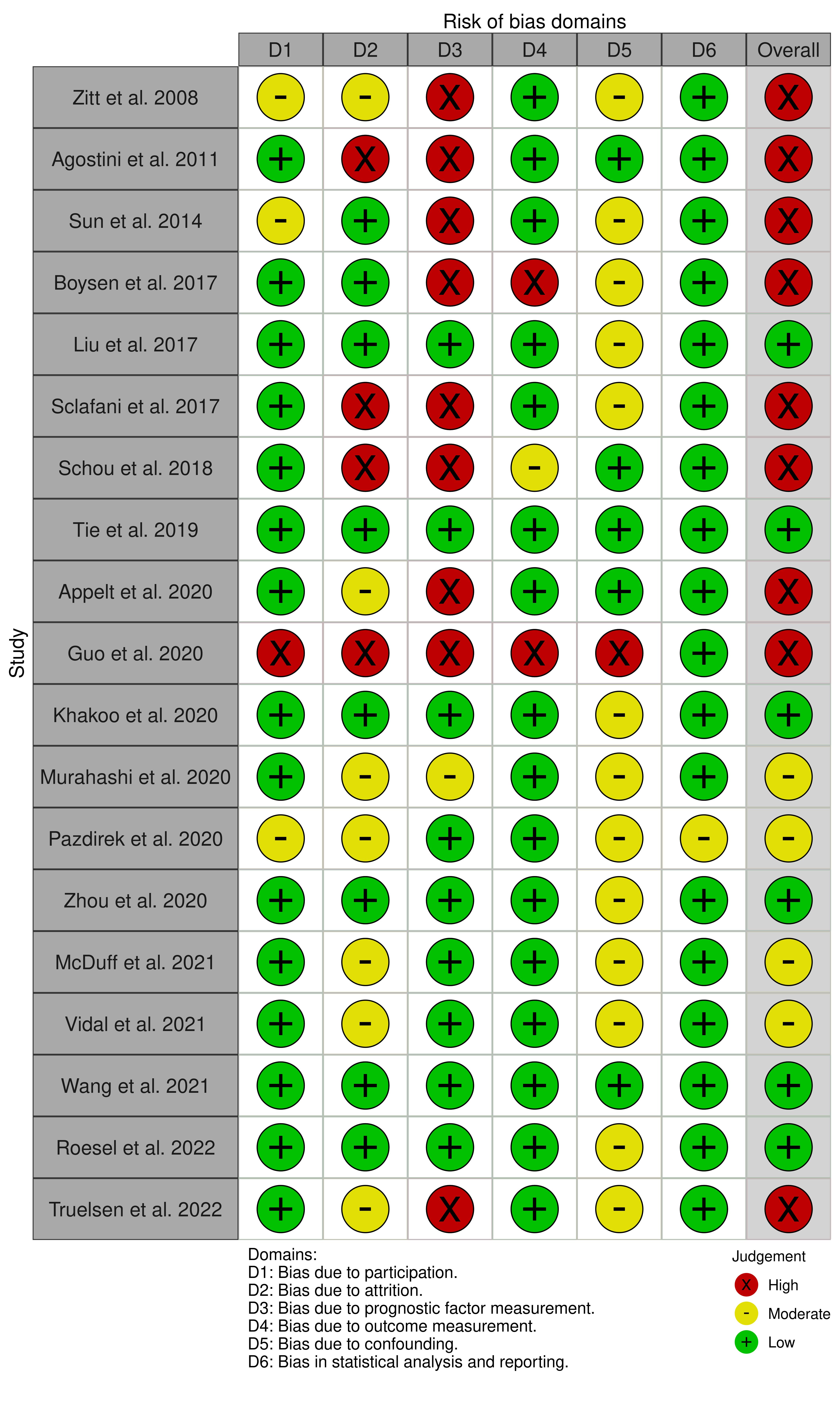


*Supplementary 3 – Reasons of bias QUIPS tool*


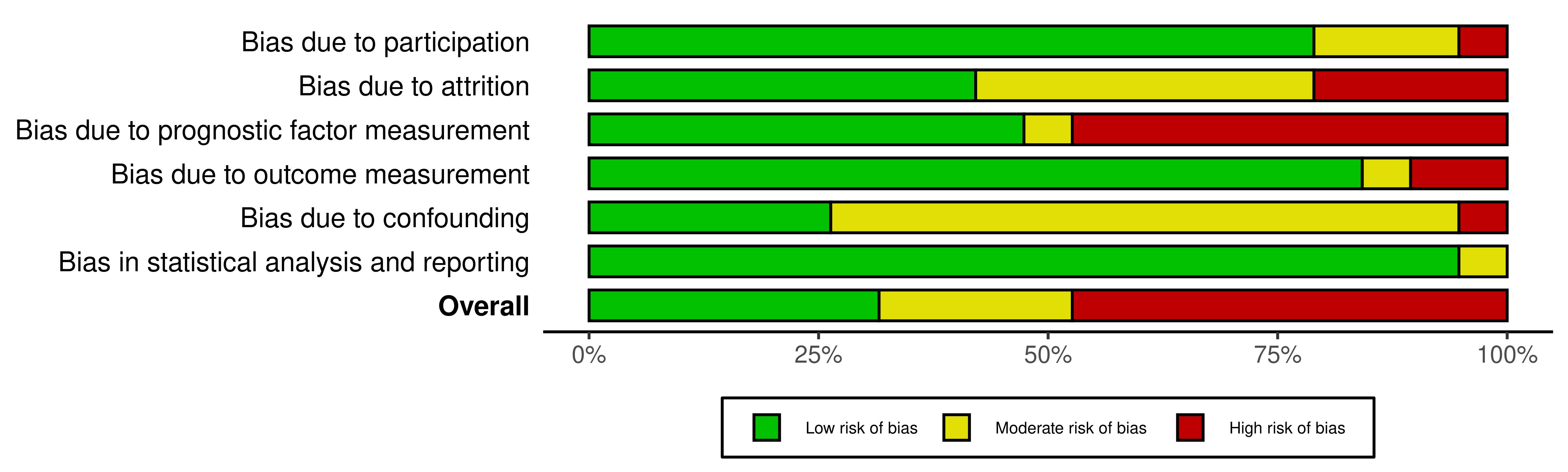


*Supplementary 4 – ctDNA measurement techniques (next page)*

| Author, year | Assay type | Target / assay | Tumour informed | Tube type | Plasma isolation | cfDNA isolation | Preprocessing | NSG / PCR | Risk of Bias |
| --- | --- | --- | --- | --- | --- | --- | --- | --- | --- |
| Zitt et al. 2008 | cfDNA concentration | cfDNA concentration (18S gene) | Agnostic | EDTA | 2x3000rpm, within 2 hours | qiaAMP | Not optimal | PCR | High |
| Agostini et al. 2011 | cfDNA concentration | cfDNA (Alu 247, Alu 115 repeat, and Alu 247/115 ratio (cfDNA integrity index)) | Agnostic | EDTA | 1x3000g, within 4 hours | qiaAMP | Not optimal | PCR | High |
| Sun et al. 2014 | Multiple | cfDNA concentration, KRAS mutation and O6-methylguanine-DNA methyltransferase promoter methylation status of cfDNA | Agnostic | Unknown | Unknown | qiaAMP | Unknown | PCR | High |
| Boysen et al. 2017 | cfDNA concentration | cfDNA concentration (beta-2 microglobulin) | Agnostic | EDTA | 1x 30g, time unknown | qiaAMP | Not optimal | PCR | High |
| Liu et al. 2017 | Mutation-specific panel | Personalised assay targeting tumour-informed mutations, universal panel of genes frequently mutated in colorectal cancer, and low depth sequencing for copy number alterations | Both | EDTA | 1x 4000g + 1x12000g, within 2 hours | Apostle MiniMax cfDNA isolation kit | optimal | NGS | Low |
| Sclafani et al. 2017 | Mutation-specific panel | KRAS/BRAF mutations | Tumour informed (predefined panel) | Unknown | Unknown | Unknown | Unknown | PCR | High |
| Schou et al. 2018 | cfDNA concentration | cfDNA concentration | Agnostic | Citrate | 1x2000g, within 2 hours | None | Not optimal | dFA | High |
| Tie et al. 2019 | Mutation-specific panel | Personalised assays | Tumour informed (tumour specific) | EDTA | 1x1200g + 1x1800g, time unknown | qiaAMP | Not optimal | NGS | Low |
| Appelt et al. 2020 | cfDNA concentration | Meth-ctDNA | Agnostic | Serum | Serum | qiaSymphony | Serum is not optimal for cfDNA analyses | PCR | High |
| Guo et al. 2020 | Promoter genes | Promoter profiling of cfDNA | Agnostic | Unknown | Unknown | qiaAMP | Unknown | NGS | High |
| Khakoo et al. 2020 | Mutation-specific panel | Personalised assays based on six oncogenes | Tumour informed (tumour specific) | Streck | 2x1600g | qiaAMP | Not optimal | PCR | Low |
| Murahashi et al. 2020 | Mutation-specific panel | cfDNA panel covering 14 genes with over 240 hotspots. | Agnostic | EDTA | 1x1600g + 1x16000g, time unknown | MagMAX | Unknown | NGS | Moderate |
| Pazdirek et al. 2020 | Mutation-specific panel | Panel of six selected oncogenes | Tumour informed (predefined panel) | Unknown | Unknown | Nucleospin plasma XS kit | Unknown | PCR | Moderate |
| Zhou et al. 2020 | Mutation-specific panel | Personalised assays | Tumour informed (tumour specific) | EDTA | 1x2500g + 1x16000g, within 3 hours | qiaAMP | Optimal | NGS | Low |
| McDuff et al. 2021 | Mutation-specific panel | Personalised assays determined by NGS | Tumour informed (tumour specific) | Streck | 1x1600g + 1x3000g | qiaAMP | Optimal | PCR | Moderate |
| Vidal et al. 2021 | Mutation-specific panel | Somatic mutations and epigenomic signatures | Agnostic | EDTA | 1x3200 rpm, within 3 hours | Unknown | Not optimal | NGS | Moderate |
| Wang et al. 2021 | Mutation-specific panel | 422 cancer-related genes | Tumour informed (predefined panel) | EDTA | 1x1800g, within 2 hours | Nucleospin plasma XS kit | Not optimal | NGS | Low |
| Roesel et al. 2022 | Mutation-specific panel | Oncomine panel | Tumour informed (predefined panel) | Streck | unknown | MagMAX | Unknown | NGS | Low |
| Truelsen et al. 2022 | cfDNA concentration | Median cfDNA | Agnostic | EDTA | 1x1200g, within 1 hours | None | Not optimal | dFA | High |

cfDNA: cell-free DNA, ctDNA: circulating tumour DNA, , dFA: direct fluorescence assay NSG: next generation sequencing, PCR: polymerase chain reaction
